# Correlation between article download and citation figures for highly accessed articles from five open access oncology journals

**DOI:** 10.1186/2193-1801-2-261

**Published:** 2013-06-13

**Authors:** Carsten Nieder, Astrid Dalhaug, Gro Aandahl

**Affiliations:** Department of Oncology and Palliative Medicine, Nordland Hospital, Bodø, 8092 Norway; Institute of Clinical Medicine, Faculty of Health Sciences, University of Tromsø, Tromsø, 9038 Norway

**Keywords:** Oncology research, Oncology bibliometrics, Publication pattern, Open access

## Abstract

Different approaches can be chosen to quantify the impact and merits of scientific oncology publications. These include source of publication (including journal reputation and impact factor), whether or not articles are cited by others, and access/download figures. When relying on citation counts, one needs to obtain access to citation databases and has to consider that results differ from one database to another. Accumulation of citations takes time and their dynamics might differ from journal to journal and topic to topic. Therefore, we wanted to evaluate the correlation between citation and download figures, hypothesising that articles with fewer downloads also accumulate fewer citations. Typically, publishers provide download figures together with the article. We extracted and analysed the 50 most viewed articles from 5 different open access oncology journals. For each of the 5 journals and also all journals combined, correlation between number of accesses and citations was limited (r = 0.01-0.30). Considerable variations were also observed when analyses were restricted to specific article types such as reviews only (r = 0.21) or case reports only (r = 0.53). Even if year of publication was taken into account, high correlation coefficients were the exception from the rule. In conclusion, downloads are not a universal surrogate for citation figures.

Comparable to other medical specialties, the field of oncology has developed rapidly over the last decades. The number of scientific publications continues to increase, and so does the number of available publication channels and methods. Open access journals have gained increasing popularity, but traditional well established high impact journals continue to attract important research and landmark clinical trials (Young et al. 2008;Stringer et al. 2008). Such research is likely to accumulate a high number of citations in the years to follow (Owlia et al. 2011). We have recently analysed pattern of citation for different fields of oncology in order to identify research mainstreams and advances, and to review the most influential preclinical and clinical developments (Nieder, 2012;Nieder et al. 2012b;2012a). Citation figures might be obtained from different sources, with known inconsistency from one to another (Durieux & Gevenois, 2010). Previous studies suggested more or less strong correlations between article download and citation figures for several areas of scientific research (Watson, 2009;Schloegl & Gorraiz, 2010). We hypothesised that oncology might follow the same traits and that frequently downloaded articles eventually also accumulate more citations than less frequently viewed articles. If true, download figures obtained from a journal's homepage might be less error prone and earlier available than citation counts, which often peak after considerable lag time. Initially we looked at the first author's own open access publications from the time period 2006–2011. For 12 articles with available access figures, a high correlation coefficient was identified (r = 0.87, p < 0.001) and therefore we decided to embark on a larger and more detailed analysis, which included several open access journals covering either all aspects or specific areas of cancer research and treatment.

## Methods

We analysed 5 BioMed Central (BMC) open access oncology journals: BMC Cancer, Molecular Cancer, Radiation Oncology, Journal of Hematology and Oncology, and World Journal of Surgical Oncology. For each of these, the 50 most viewed articles of all time irrespective of category or topic were selected from the journals' homepage (fields "most viewed" and "all time", accessed on January 23rd, 2013). We restricted the present analysis to BMC journals because we wanted to rule out the possibility that different publishers use different methods to define their most viewed articles, which could result in systematic errors. On the same day, citation numbers were obtained for all these articles by accessing the abstract and citation database Scopus (Elsevier B.V., http://www.scopus.com). Correlations were first analysed for each journal separately, then for specific article types such as review, case report and research article within each journal. Due to insufficient numbers (4 meta-analyses, 1 randomised clinical trial), these two types of research could not be evaluated in detail. Analyses stratified by article type were also performed for the combined data set from all 5 journals. Furthermore, analyses stratified by year of publication were performed. For all statistical analyses, Pearson's correlation coefficient was computed.

## Results

Table 1 shows basic information about all 5 journals (incl. number of articles published during each given year and current impact factor) and detailed information about the most viewed (so called top 50) articles from each journal. The highest number of articles was published in BMC Cancer, which also is the oldest journal and the only one to cover the whole field of oncology. Each of the 5 journals contained less than 50 articles in their first volume, with marked increase in publications either during the fourth or fifth year, but no linear growth afterwards. These figures probably reflect the visibility and reputation of new journals, which might become more attractive when they receive their first impact factor and with increasing impact factor over time.

**Table 1:** **Publication data from 5 open access oncology journals (50 most often viewed articles)**

	Radiation oncology	Journal of hematology and oncology	World journal of surgical oncology	BMC cancer	Molecular cancer
Impact factor (2011)	2.32	3.99	1.12	3.01	3.99
First volume published (year)	2006	2008	2003	2001	2002
Number of top 50 publications 2001				3 (20)	
Number of top 50 publications 2002				5 (37)	2 (9)
Number of top 50 publications 2003			11 (30)	4 (33)	14 (42)
Number of top 50 publications 2004			12 (47)	9 (98)	5 (38)
Number of top 50 publications 2005			9 (78)	2 (164)	3 (43)
Number of top 50 publications 2006	12 (48*)		7 (97)	10 (298)	10 (76)
Number of top 50 publications 2007	16 (45)		4 (146)	3 (237)	6 (83)
Number of top 50 publications 2008	9 (44)	11 (27)	2 (139)	6 (396)	1 (94)
Number of top 50 publications 2009	8 (71)	23 (51)	4 (102)	4 (465)	3 (133)
Number of top 50 publications 2010	3 (122)	9 (51)	1 (114)	2 (697)	4 (320)
Number of top 50 publications 2011	2 (182)	7 (54)	0 (174)	2 (529)	2 (152)
Number of top 50 publications 2012	0 (226)	0 (75)	0 (280)	0 (627)	0 (91)
Number of case reports in top 50	1	9	19	3	0
Number of review articles in top 50	6	30	19	2	24
Median number of accesses, range	9056.5, 22329-7094	7212, 24207-5375	18178, 91411-12184	13308.5, 43299-10786	18313, 50383-12598
Median number of citations, range	16.5, 0-71	12, 0-114	15.5, 1-53	28.5, 3-214	60.5, 4-582

*Number in parentheses: total number of articles published during the same year.

**Figure 1:**
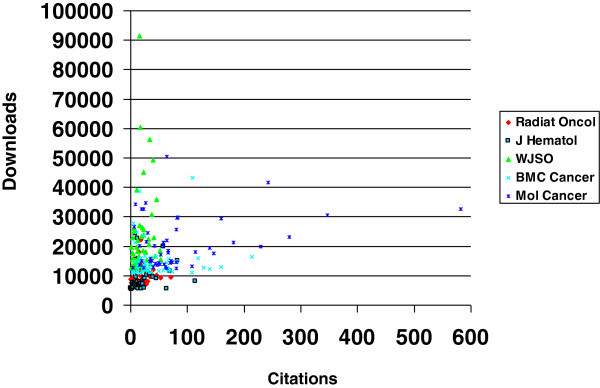
**Correlation between number of accesses and citations for 5 open access oncology journals (50 most viewed articles of all time for each journal).**

None of the articles published during the year 2012 was among the top 50 viewed publications, and very few articles published during the year 2011 were on this list. Among all top 50 articles, the one that attracted most readers had 91411 accesses, the median number varied from journal to journal and ranged between 7212 and 18313 (Table 1). The number of citations ranged between 0 and 582. The median number varied from journal to journal and ranged between 12 and 60.5. Even if median number of accesses was comparable between two journals (approximately 18000 for the World Journal of Surgical Oncology and Molecular Cancer), median number of citations was not (15.5 vs. 60.5, p < 0.001). When analysed separately, correlation between number of accesses and citations was poor to moderate for each of the journals (r = 0.01-0.30, Table 2 and Figure 1). The same was true for articles from all 5 journals combined (r = 0.23). Since both variables are time-dependent, we analysed the data on a year by year basis. For all journals combined, the highest correlation was found for articles published during the year 2011 (r = 0.47). Other figures included −0.03 for 2010, 0.18 for 2009 and 0.01 for 2008. When analysed for each journal separately, considerable differences were found. Looking for example at the data from 2009, a correlation coefficient of 0.67 was seen for the Journal of Hematology and Oncology (all articles regardless of research type). For the World Journal of Surgical Oncology this figure was 0.44, for Molecular Cancer 0.17, for Radiation Oncology 0.01 and for BMC Cancer −0.77.

**Table 2:** **Results overview: correlation coefficients**

	All 5 journals	Radiation oncology	Journal of hematology and oncology	World journal of surgical oncology	BMC cancer	Molecular cancer
All top 50 articles	0.23	0.01	0.22	0.18	0.02	0.30
Limited to 2011	0.47	*	−0.19	*	*	*
Limited to 2010	−0.03	*	0.44	*	*	*
Limited to 2009	0.18	0.01	0.67	0.44	−0.77	0.17
Limited to 2008	0.01	0.18	−0.21	*	0.22	*
All case reports	0.53	*	0.54	0.52	*	*
Case reports 2011	*	*	*	*	*	*
Case reports 2010	*	*	*	*	*	*
Case reports 2009	*	*	*	*	*	*
Case reports 2008	*	*	*	*	*	*
All reviews	0.21	−0.28	0.25	−0.09	*	0.22
Reviews 2011	0.41	*	−0.25	*	*	*
Reviews 2010	0.09	*	0.23	*	*	*
Reviews 2009	0.30	*	0.78	*	*	*
Reviews 2008	−0.57	*	*	*	*	*
All research articles	0.34	0.08	0.06	0.51	0.10	0.55
Research articles 2011	*	*	*	*	*	*
Research articles 2010	−0.42	*	*	*	*	*
Research articles 2009	−0.13	0.02	*	*	*	*
Research articles 2008	0.27	0.05	*	*	0.22	*

*Not calculated because 5 or less articles available for analysis.

Whether or not correlations exist might also depend on type of research. Regarding all review articles (n = 81, all journals, all years of publication), a value of 0.21 was found. Even if limited to each specific journal, a maximum of 0.25 was observed. Stratified by year of publication, the highest correlation was found for reviews published during the year 2011 (r = 0.41). When stratified by year of publication and journal, reviews published in the Journal of Hematology and Oncology during the year 2009 achieved a correlation coefficient of 0.78 whereas those published in Molecular Cancer during the year 2003 achieved −0.08. Compared to all 81 review articles (correlation coefficient 0.21), better results were seen for case reports (n = 32, all journals, all years of publication) with a correlation coefficient of 0.53, and for research articles (n = 225, all journals, all years of publication) with a correlation coefficient of 0.34.

## Discussion

The present analysis focussed on highly accessed articles published in 5 arbitrarily selected open access oncology journals. It revealed interesting differences between these journals (0–19 case reports among the top 50 articles, 2–30 reviews), for example related to access and citation numbers. For each of the 5 journals and also all journals combined, the correlation between number of accesses and citations was poor to moderate (r = 0.01-0.30). Considerable variations were also observed when these analyses were restricted to specific article types such as reviews only (r = 0.21), research articles only (r = 0.34) or case reports only (r = 0.53). Even if year of publication was taken into account, high correlation coefficients were the exception from the rule. The following example illustrates these findings. Reviews published in the Journal of Hematology and Oncology during the year 2009 achieved a correlation coefficient of 0.78 whereas those published in Molecular Cancer during the year 2003 achieved −0.08. These results were surprising and in contrast to our expectations and initial findings from a preliminary analysis of the first author's open access publications (correlation coefficient 0.87). Possibly, correlations become weaker when analyses are focused on highly viewed articles rather than all articles published in a given open access journal. The interest of the readers (a heterogeneous group including for example practising oncologists, scientists, technicians, nurses, students and patients; open access without institutional subscription or fees) might not necessarily reflect the scientific impact of a given topic or practical implications of an unusual case, and the likelihood of citation in other articles (Kanaan et al. 2011). Citation frequency is also dependent on other factors including but not limited to number of authors and contributing institutions (Figg et al. 2006;Stringer et al. 2010). We are not aware of other analyses limited to the most viewed articles.

Potential limitations of the present study, aside from limiting the analyses to the most viewed articles, include the low numbers of articles in the different categories and years, and the low number of oncology journals, which are not fully representative of the broad field of cancer causes, epidemiology, research and treatment with all its different subspecialties.

Paiva et al. evaluated open access journals from the BMC and Public Library of Science (PLoS) publishing groups (all 6 PLoS journals, as well as the 6 best ranked and the 6 worst ranked BMC journals, according to Journal Citation Reports (JCR) 2010) (Paiva et al. 2012). None of the journals analysed in the present study was included. All original research articles published from September 1, 2008, to September 30, 2008, were analysed (not limited to oncology). Articles classified as review articles, case reports, commentaries, editorials, and letters to the editor were excluded from the analysis. The three-year period spanning from the article publication to the time of analysis was considered to be a sufficient amount of time to measure the impact of a specific article in the scientific community. The numbers of times the article was viewed at the publisher site, downloaded, and cited according to JCR Science Edition 2010 were collected for the period from December 6, 2011, to December 20, 2011. In total, 423 original research article titles were included in the analysis. The median number of views and citations were 2533 and 10, respectively (fewer than our data derived from top 50 publications). There was a positive correlation between the number of views and citations (r = 0.434, p < 0.001).

For the Journal of Vision (free access), comparable evaluations were performed (Watson, 2009). One comparison was between the total downloads and total citations. The correlation between these two quantities was 0.74, indicating a strong positive relationship. To neutralize the growth with age, they compared the total downloads and citations (as of July 1, 2008) for papers published in a given year. There was a strong positive correlation in each year, with a high of around 0.8 in 2003. Because of the lag between downloads and citations, one would not expect correlations to be as high for articles less than three years old. In articles at least three years old, the correlation was always above 0.6 (except for 2001, which was based on only 12 articles). This analysis indicated that download statistics provide a useful indicator, two years in advance, of eventual citations.

In contrast to these two studies, Schloegl and Gorraiz looked at oncology journals only (Schloegl & Gorraiz, 2010). None of the journals analysed in the present study was included. They identified a strong correlation between the citation frequencies and the number of downloads for their journal sample. The relationship was lower when performing the analysis on a paper by paper basis because of existing variances in the citation-download-ratio among articles. They computed Spearman rank correlation coefficients of 0.89 and twice 0.92 between the 2004 downloads and the particular 2004, 2005 and 2006 cites. The corresponding correlations between the downloads and citations of the years 2005 (n = 31) and 2006 (n = 33) were similar (between 0.9 and 0.92). Because of the big differences between downloads and citations especially in the publication year, a high correlation was not expected in 2006 (for instance 0.32 for Cancer Letters and 0.41 for Gynecologic Oncology).

Our own results derived from other oncology journals than those evaluated previously suggest that complex and variable relations exist between downloads and citations. We can not recommend a universal strategy that substitutes citation figures by downloads for the purpose of quantitative analyses.
